# Identifying factors associated with the hospital readmission rate among patients with major depressive disorder

**DOI:** 10.1186/s12888-021-03559-7

**Published:** 2021-11-01

**Authors:** Sharareh Parami, Leili Tapak, Jalal Poorolajal, Abbas Moghimbeigi, Ali Ghaleiha

**Affiliations:** 1 Department of Biostatistics, School of Public Health, Hamadan University of Medical Sciences, Hamadan, Iran; 2 Modeling of Noncommunicable Diseases Research center, Hamadan University of Medical Sciences, Hamadan, Iran; 3 Department of Epidemiology, School of Public Health, Hamadan University of Medical Sciences, Hamadan, Iran; 4 Research Center for Health Sciences, Hamadan University of Medical Sciences, Hamadan, Iran; 5 Department of Biostatistics, School of Public health, Alborz University of Medical Sciences, Alborz, Iran; 6 Department of Psychiatry, School of Medicine, Hamadan University of Medical Sciences, Hamadan, Iran; 7 Behavioral Disorders and Substance Abuse Research Center, Hamadan University of Medical Sciences, Hamadan, Iran

**Keywords:** Count model, Generalized Poisson, Zero-inflated, Hospitalization, Major depressive disorder

## Abstract

**Background:**

Major depressive disorder (MDD) is a common recurrent mental disorder and one of the leading causes of disability in the world. The recurrence of MDD is associated with increased psychological and social burden, limitations for the patient, family, and society; therefore, action to reduce and prevent the recurrence of this disorder or hospital readmissions for depression among the patients is essential.

**Methods:**

The data of this retrospective cohort study were extracted from records of 1005 patients with MDD hospitalized in Farshchian hospital in Hamadan city, Iran (2011–2018). The hospital readmissions rate due to depression episodes was modeled using generalized Poisson regression (GPR). Demographic and clinical characteristics of the patients were considered as explanatory variables. SAS v9.4 was used (*P* < 0.05).

**Results:**

A majority of the patients were male (66.37%). The mean (standard deviation) of age at onset of MDD and the average number of hospital readmissions were 32.39 (13.03) years and 0.53 (1.84), respectively (most patients (74.3%) did not experience hospital readmissions). According to the results of the GPR, the lower age at the onset of the disease (IRR = 1.02;*P* = 0.008), illiteracy (IRR = 2.06;*P* = 0.003), living in urban areas (IRR = 1.56;*P* = 0.015), history of psychiatric illnesses in the family (IRR = 1.75;*P* = 0.004), history of emotional problems (IRR = 1.42;*P* = 0.028) and having medical disorders (IRR = 1.44;*P* = 0.035) were positively associated with the number of hospitalizations.

**Conclusion:**

According to our findings, urbanization, early onset of the disease, illiteracy, family history of mental illness, emotional problems, and medical disorders are among major risk factors associated with an increased number of hospital readmissions of MDD.

**Supplementary Information:**

The online version contains supplementary material available at 10.1186/s12888-021-03559-7.

## Introduction

Major depression is a common mental disorder and one of the leading causes of disability and the burden of diseases in the world. About 300 million people are affected by depression worldwide and there was an increase of more than 18% between 2005 and 2015 [1]. Depression is the leading cause of early retirement and absence from work due to illnesses in many European countries [2]. Depression is very common in people with chronic diseases such as diabetes, heart disease, rheumatoid arthritis, kidney disease, the acquired immunodeficiency syndrome (AIDS), and multiple sclerosis, and studies have shown that about one-third of these patients suffer from depression [3].

According to the World Health Organization (WHO), depression is the second most important and serious disorder after cardiovascular disease and ranks fourth among health problems. Depression is expected to impose a heavy burden on communities by 2020 [4]. Depression can be long-term and recurrent, significantly impairing the ability of people to work at school or in everyday life and feel that life is worthless [5–7]. This significant violation of daily functioning is equal to or greater compared to other chronic medical conditions such as diabetes and congestive heart failure [8]. As a result, MDD can have a significant impact on the quality of life and work productivity [9, 10]. The average onset age of major depressive disorder (MDD) is around forty, in which approximately 50% of the cases begin between the ages of 20 and 50 [11]. Some new epidemiological data suggest that the incidence of MDD may be increasing among people under 20 [12, 13].

MDD is both a chronic and recurrent disease. Recurrence after recovery is common in MDDs [13]. Patients who are at least in their second period of depression are in the recurrent type of MDD. Although relieving the signs and symptoms of depression is the main goal of treatment, maintaining this state of health is one of the current challenges in mental health. Both natural and therapeutic studies of MDD have highlighted the high rate of recurrence after recovery [14–19]. Persistence or recurrence is the most important clinical problem associated with depression that leads to significant consequences for the individual and general health [20, 21]. The higher the number of previous courses, the faster the recurrence, bringing about not only a shorter interval between cases but also a greater intensity of each one [15, 17, 18]. As studies have shown, approximately 50% of those who recover from the first MDD would experience another period in their lifetime, roughly 80% of those with a history of two periods would relapse [20, 22–26], and finally, on average, each depressed patient experiences 3 to 4 periods of depression in his or her lifetime [27]. Gender, level of education, employment status, and marital status are all the probable risk factors that may lead to a recurrence in a depressed patient [28–32]. One of the goals of the WHO in promoting mental health and treatment of mental illness is to reduce the duration and number of hospitalizations and also to expand social services, which can reduce the psychological, emotional, economic, and social burden and improve physical health and quality of life of family members [33, 34]. Therefore, it is necessary not only to identify risk factors of depression but also to deal with them [35].

Studying reversible diseases, identifying the factors affecting their recurrence or hospital readmission, and finally preventing them are all among the most important issues in medicine. Since MDD is a recurrent disease and its recurrence or hospital readmission is associated with increased psychological, social, and other burdens that the patients and their family are faced with, the use of appropriate statistical methods to model the recurrence of the disease has a significant role in identifying the factors affecting it. Most studies on recurrent depression have used logistic regression to model recurrent as a binary response, so that the absence/presence of a single episode of the disease is modeled [25, 36]. This approach is faced with limitations because it neglects the rate of recurrent episodes as a crucial outcome. In this regard, considering outcomes like the number of re-hospitalizations or readmissions due to the disease or the degree of recurrence as count outcomes (that somehow reflects the recurrent rate) and its modeling are of particular importance. This is especially important because MDD has a highly recurrent nature and its recurrence varies across individuals substantially [37].

Due to the high cost of treatment, maintenance, and the costs imposed by crimes caused by depression disorder, its damages cannot be calculated as it affects all aspects of patients’ lives and functions negatively and often requires long-term care and follow-up. It has also been shown that the incidence of MDD may be increasing among people aged under 20. This study aimed to compare different count regressions in modeling hospital readmissions due to MDD and to investigate the factors associated with MDD in Hamadan province. Our results would help to control the disease and to prevent recurrence or hospital readmission in these patients in Hamadan province.

## Materials and methods

### Data

In this retrospective cohort study, information of all 1005 patients with MDD admitted to Sina (Farshchian) Educational hospital (the referral hospital and the only psychiatric hospital across Hamadan Province) in Hamadan, Iran was extracted from their records from 2011 to 2018. In this study, only the patients who were admitted for treatment of depression are considered. MDD was diagnosed through a clinical interview which was conducted by a psychiatrist based on Diagnostic and Statistical Manual of Mental Disorders (DSM-5) [13]. Data were collected using a checklist prepared by the authors and clinical consultant. The checklist included demographic characteristics and clinical characteristics of the patients. The information related to the explanatory variables (recorded in the patients’ cases) had been recorded by a medical doctor based on patients’ and their companions’ statements.

#### The response variable (outcome)

The number of hospital readmissions from the beginning of the disease to the end of the study (2011–2018) of patients with MDD was considered as the response variable. As the Sina hospital was the only place for admitting the patients with major depression for hospitalization throughout the Hamadan province, most of the patients were hospitalized only in this place. However, records of hospital readmissions in places outside of the province were also extracted and pooled with the response variable.

#### Explanatory variables

Information was used as explanatory variables as follows: 1) Demographic characteristics including gender (male/female), marital status (single (including never married, divorced, widow (er))/married), education level (illiterate/literate), occupation (unemployed/employed), type of residence (city/village), the number of siblings, history of emotional problems including death/disease of a family member or relatives, divorce of parents, marriage, pregnancy, having a new member in the family, an emotional failure (breaking up with a girlfriend or boyfriend, breaking up the engagement or marriage, divorce, infidelity, etc.), having a girl or boy abandoning the house and military service (yes/no); 2) Variables related to patients’ clinical characteristics including age at the onset of the disease, history of psychiatric illnesses/mental disorders in family members (yes/no), history of medical disorders including cardiovascular, visual and auditory diseases, skin problems, hypertension, hyperlipidemia, glands (including thyroid and diabetes), gastrointestinal problems (including gastric ulcer and pancreatitis), neurological problems including stroke, headache and seizures), rheumatic problems (including lung and asthma) and urological problems (including kidney and urinary tract diseases), having a history of suicide attempt (yes/no), having a history of smoking (yes/no), having a history of substance abuse including opium, cannabis, glass and psychotropic substances, etc., (yes/no). All variables were selected based on previous studies and information available. A description of the selected explanatory variables was provided in Table 1.

**Table 1:** Demographic and personal characteristics of the college patients

Covariates	Levels	Number (Percent)	Number of recurrences Mean ± SD
**Gender**	Male	(66.37) 667	0.47 ± 1.67
	Female	(33.63) 338	0.66 ± 2.12
**Marital status**	Single	(32.84) 330	0.47 ± 2.11
	Married	(67.16) 675	0.56 ± 1.69
**Educational level**	Illiterate	(16.22) 163	0.89 ± 2.32
	Literate	(83.80) 842	0.47 ± 1.73
**Occupation**	Unemployed (housewife/vacant)	(60) 603	0.61 ± 1.80
	Employed	[40] 402	0.43 ± 1.90
**Type of residence**	Urban	(70.15) 705	0.60 ± 2.09
	Rural	(29.85) 300	0.39 ± 1.01
**Number of siblings**	0–1	(5.27) 53	0.28 ± 0.57
	2–3	(17.31) 174	0.60 ± 2.92
	< 4	(77.41) 778	0.54 ± 1.84
**History of emotional problems**	No	(39.6) 398	0.40 ± 1.10
	Yes	(60.4) 607	0.62 ± 2.19
**History of smoking**	No	(39.6) 398	0.53 ± 1.09
	Yes	(60.4) 607	0.54 ± 2.20
**History of substance abuse**	No	(46.87) 471	0.6 0 ± 1.57
	Yes	(53.13) 534	0.48 ± 2.05
**Age of onset (year)**	> 20	(19.2) 193	0.63 ± 2.62
	20–29.99	(29.45) 296	0.51 ± 1.72
	30–39.99	(23.48) 236	0.53 ± 1.93
	< 40	(27.86) 280	0.51 ± 1.09
**Family history of psychiatric illness**	No	(78.31) 787	0.45 ± 1.43
	Yes	(21.69) 218	0.82 ± 2.86
**History of medical disorders**	No	(58.8) 591	0.42 ± 1.64
	Yes	(41.2) 414	0.70 ± 2.09
**History of medication nonadherence**	No	(58.41) 587	0.47 ± 1.30
	Yes	(41.59) 418	0.58 ± 2.14

### Statistical analysis

The hospitalization rate among patients with MDD was modeled as a function of gender, age of onset, and other explanatory variables using several count regression models including Poisson, negative binomial (NB), generalized Poisson (GP), exponentiated-exponential geometric (EEG), and their zero-inflated (ZI) counterpart regressions (Please see the Additional file 1 for model desciption). All variables were the same considered in all models. Vuong test based on Bayesian Information criteria (BIC) was used to compare non-nested models (All tests were conducted with α ≤ 0.05) to see which one provides a better fit. This test produces a z-statistic where a value > 1.96 supports the alternative that the first model fits the data better and a value <− 1.96 indicates the second model fits better [38]. Bonferroni correction was utilized for multiple comparisons. For details of other count regression models (Poisson, NB, GP, EEG and Zero-Inflated models) see [39]. Exponentiated coefficients (incidence rate ratio (IRR)) and 95% confidence interval were estimated for each model.

#### Software

All analyses were conducted using R.3.6.2. and SAS 9.2.

## Results

In this study, information of about 1005 patients with MDD in Farshchian hospital Hamadan was used. About 74% (747 out of 1005 patients) did not experience rehospitalization and the mean number of hospitalizations was 0.53 (SD = 1.84). Table 1 shows the characteristics of the patients in this study. According to the results shown in Table 1, 66.4% of patients were male. The mean age of patients was 39.53 years (SD = 13.23) with the majority over 45 years (35%). Most patients in the study were married (67.2%). Most patients (83.8%) were literate. About 60% of the patients were unemployed (housewives and unemployed); the majority of them lived in the city (70.2%). Most patients had 4 or more siblings (70.4%), 60.4% had a history of emotional problems, 60.4% had a history of smoking, and about 53% had a history of substance abuse. The mean age of onset was 32.39 years (SD = 13.03) with the majority in the age group over 40 years (27.9%). 21.7% of patients had a family history of psychiatric illness and 41.2% had a history of medical disorders, 41.6% of patients had a history of medication nonadherence.

Table 2 shows the results of the Vuong test related to the fitting of different models including Poisson, zero-inflated Poisson (ZIP), NB, zero-inflated negative binomial (ZINB), GP, zero-inflated generalized Poisson (ZIGP), EEG, and zero-inflated exponentiated-exponential geometric (ZIEEG) to MDD data. The provided results of the Vuong test were calculated based on BIC. According to the Voung test statistic (Bonferroni correction was considered), the negative binomial and generalized Poisson regression were the best cases among all regression models. There was no significant difference between models of NB and GP, therefore we selected GP with smaller Bayesian Information criteria (BIC = 1876.2).

**Table 2:** The results of the Vuong test for pairwise goodness of fits of different distributions over major depression dataset

Vuong-BIC	Poisson	NB	GP	EEG	ZIP	ZINB	ZIGP
**ZIEEG**	2.616	−7.256^a^	− 7.460^a^	−9.254^a^	2.577	−2.443	−2.925^a^
**ZIGP**	2.648	−4.046^a^	−6.966^a^	−2.199	2.574	2.882^a^	
**ZINB**	2.630	−6.864^a^	−8.484^a^	−4.217^a^	2.543		
**ZIP**	2.420	−3.325^a^	−3.283^a^	− 3.315^a^			
**EEG**	2.998^a^	−3.191^a^	−2.722				
**GP**	3.006^a^	2.315					
**NB**	3.012^a^						

^a^Significant at 0.002 (Bonferroni correction was considered); negative binomial (NB), generalized Poisson (GP), exponentiated-exponential geometric (EEG), and their zero-inflated (ZI) counterparts

So, overall, the GP model was selected here as the final model.

Table 3 shows the regression coefficients of the GP regression model, which is proportional to the number of hospitalizations of patients with MDD. According to the results shown in Table 2, the variables that were significantly associated with the number of hospitalizations included lower age of onset of the disease (IRR = 1.02; 95% CI: 1.01, 1.03; *P* = 0.008), illiteracy (IRR = 2.06; 95% CI: 1.28, 3.33; *P* = 0.003), living in urban areas (IRR = 1.56; 95% CI: 1.09, 2.22; *P* = 0.015), having a family history of psychiatric illness (IRR = 1.72; 95% CI: 1.19, 2.48; *P* = 0.004), having a history of medical disorders (IRR = 1.44; 95% CI: 1.04, 1.99; *P* = 0.028), having a history of emotional problems (IRR = 1.42; 95% CI: 1.03, 1.98; *P* = 0.035).

**Table 3:** Regression coefficients obtained from the multivariate generalized Poisson regression model for major depression data

Covariates	Levels	β (SE)	t-value	***P***-value	Exp(β)	95% Confidence Limits	
**Intercept**		−0.016 (0.47)	−0.03	0.973	0.984	0.39	2.46
**Gender**	Male	Reference category					
	Female	−0.047 (0.21)	− 0.22	0.827	0.954	0.63	1.45
**History of medication nonadherence**	No	Reference category					
	Yes	0.129 (0.16)	0.79	0.43	1.138	0.83	1.57
**Age of onset**		−0.02 (0.01)	−2.68	0.008^b^	0.98	0.97	0.99
**Occupation**	Unemployed	Reference category					
	Employed	−0.28 (0.18)	−1.55	0.121	0.756	0.53	1.08
**Number of siblings**		−0.008 (0.04)	−0.23	0.82	0.992	0.92	1.07
**Educational level**	Illiterate	Reference category					
	Literate	−0.723 (0.24)	−3.01	0.003^b^	0.486	0.3	0.78
**Type of residence**	Urban	Reference category					
	rural	−0.447 (0.18)	−2.44	0.015^a^	0.639	0.45	0.92
**Marital status**	Single	Reference category					
	Married	0.308 (0.19)	1.59	0.112	1.361	0.93	1.99
**Family history of psychiatric illness**	No	Reference category					
	Yes	0.543 (0.19)	2.92	0.004^b^	1.72	1.19	2.48
**History of medical disorders**	No	Reference category					
	Yes	0.364 (0.17)	2.2	0.028^a^	1.439	1.04	1.99
**History of emotional problems**	No	Reference category					
	Yes	0.353 (0.17)	2.11	0.035^a^	1.424	1.03	1.98
**History of substance abuse**	No	Reference category					
	Yes	−0.315 (0.28)	−1.14	0.253	0.73	0.43	1.25
**History of smoking**	No	Reference category					
	Yes	0.316 (0.28)	1.12	0.263	1.372	0.79	2.39
**θ (**Dispersion parameter**)**		1.293 (0.13)	9.62	< 0.0001^b^			
**−2 Log Likelihood**		1772.5					
**AIC**		1802.5					
**BIC**		1876.2					

^a^Significant at 0.05; ^b^Significant at 0.01

## Discussion

Depression is one of the most common psychiatric diagnoses and its growing trend and prevalence have created a major problem for mental health. Persistence or recurrence is the most important clinical problem associated with depression that leads to significant consequences on individual and general health. This persistence or recurrence is one of the causes of significant stress depression; therefore, studying its underlying factors is of great importance. In this study, the GP regression model was found to have a better fit for the count response. Therefore, it was utilized to determine the factors related to the number of hospital readmissions of MDD among patients.

Our finding showed that men had a higher hospital readmission rate (66.4%). While considerable evidences show that there are no differences for the risk of recurrence of depression among men and women [40], it might be due to the significantly better adherence to medications among women that we observed in this study (results were not shown). Also, the prevalence of unemployment was significantly higher among men than the women in our study (results were not shown). A systematic review and meta-analysis has shown that there is a positive association between unemployment and depression and this association was higher in men compared with women (OR = 2.27 for men v.s OR = 1.62) [41].

The findings of the present study showed that the younger the age of onset of the disease, the more the average number of hospitalizations. Our findings showed that younger age of onset causes the mean number of recurrences to increase 1.02 times. Gilman et al. [37] also showed that younger age at the onset of depression reduces the likelihood of recovery.

Findings of our study showed that illiteracy leads to an increase in the average number of hospitalizations, revealing that illiteracy compared to literacy has increased the average number of hospitalizations by 2.06 times. In the studies of Gilman et al. [37] and McLeod et al. [42], a low level of education was effective in reducing the likelihood of recovery. Also, in the study of Stegenga et al. [43], a low level of education significantly increased the risk of long-term MDD with a high number of recurrences compared to single and short-term depression. In the studies of Zamanian et al. [44], and Chevalier & Feinstein [45], there was a significant relationship between a low level of education and the severity of depression.

Our findings also showed that having a history of psychiatric illness in the family leads to an increase in the average number of hospitalizations, proving that having a history of psychiatric illness in the family has increased the average number of recurrences by 1.89 times. In the study of Teraishi et al. [46], having a family history of psychiatric illness was a predictor of recurrence. The study of Burcusa & Iacono [20] also showed that having a family history of psychiatric illness increases the risk of recurrence. In the study of Skodol et al. [25], having a family history of psychiatric illness was a strong predictor of the persistence of MDD. If one parent has a mood disorder, their child has a 10 to 25% risk of developing a mood disorder. If both parents are infected, the risk doubles. The more infected people there are in the family, the greater the risk of infecting children will be [11].

Our findings also show that urbanization leads to an increase in the average number of hospitalizations. This may be due to the dramatic increase in Iran’s population over the past few decades. 70% of Iran’s population lives in urban areas and the cities faced serious problems with population growth and high unemployment. On the other hand, the stresses of urban life increase the incidence of mental illness [47]. Also, the lack of access to and distance from specialized medical services to receive services and the economic situation of the villagers causing them to visit less specialized medical centers, and as a result, they are hospitalized less often.

The findings of the present study showed that having a history of emotional problems leads to an increase in the average number of hospitalizations. Studies by Brown & Moran and Brown et al. have identified emotional problems as an important predictor of the duration of depressive episodes in depressed women [48, 49]. Stressful events such as the loss of parents and spouse, either separation or death, are the strongest predictors of the onset of periods of depression. Studies have shown that what patients believe to be stress-bringing events that hurt his or her self-esteem is more likely to cause depression. In addition, what may be considered a relatively mild stress-bringing event by others may have devastating effects on the patient because of the unique meanings it gives [13].

The findings of our study showed that having a history of medical disorders leads to an increase in the average number of hospital readmissions. In the study of Hynninen et al. [50], those with the chronic obstructive pulmonary disease had a higher risk of recurrence of depression. In the studies of Spijker et al. [51] and Geerlings et al. [52], chronic physical illness has increased the severity of MDD. Patients with medical disorders may need more daily care due to the gradual deterioration of their physical condition, which can reduce self-esteem and self-efficacy and increase the risk of MDD. Many neurological and medical disorders and many medications can cause depression symptoms [50]. In mood disorders, there is a rule that any medication taken by a sick person should be considered a potential factor in causing a mood disorder. Hence, cardiac, antihypertensives, analgesics, antibacterials, anti-cancer, and other similar drugs are commonly associated with depression symptoms [13].

In the present study, no significant relationship between gender and the number of hospital readmissions was observed which was quite similar to findings of others including Perlis et al. [53], Rucci et al. [54], Hardwell et al. [55], and Skodol et al. [25]. Similar to the studies of Onaemo et al. [56] and Skodol et al. [25], marital status, in our study also had no significant relationship with the number of hospital readmissions. It was also revealed that the variables including following specific drug prescription, drug abuse, and smoking cigarettes showed no significant relationship with the number of hospital readmissions, proving the findings of other studies done by Saragoussi et al., [36] Spijker et al. [51], and Kendler et al. [57].

Because the data were extracted from patients’ records in the present study, we faced some limitations including not taking into account some important variables such as alcohol consumption, which is significantly associated with the desired outcome. We also had some other limitations regarding the number of episodes or recurrent of major deppression as, during the research time, being 2011 to 2018, some patients did not return to Sina (Farshchian) hospital in Hamadan. This was due to either the patient changing the place of residence or not taking the hospital orders seriously. Also, there may be some patients who do not go to the doctor for follow-up treatment and hospital in the episodes or some patients who go to the doctor in episodes for outpatient treatment, change of medications and doses, and finally, patients who go to the hospital in episodes but refuse to be admitted with personal consent. These cases are beyond the control of the researcher and are uncontrollable, so they are among the limitations of the study. This was due to either the patient changing the place of residence or not taking the hospital orders seriously. The cross-sectional nature of this study was another limitation that could limit our results since the findings did not indicate any cause-and-effect relationships. Despite these limitations we considered a count variable as the response variable instead of a binary variable and tested several existing count models to select the model with a better fitting to the data.

## Conclusions

The multivariate model utilized in the present research helped to identify factors associating with the hospital readmissions rate of depressive episodes among patients with major depressive disorder. In fact, our findings showed, urbanization, early onset of the disease, illiteracy, family history of mental illness, emotional problems, and medical disorders are among major risk factors associated with an increased number of hospital readmissions of major depressive disorder.

## Supplementary Information

**Additional file 1.** Count regression models.

## Data Availability

The datasets used and/or analyzed during the current study are available from the corresponding author on reasonable request.

## Abbreviations

NB: negative binomial
GP: generalized Poisson
EEG: exponentiated-exponential geometric
ZI: zero-inflated
ZIP: zero-inflated Poisson
ZINB: zero-inflated negative binomial
ZIEEG: zero-inflated exponentiated-exponential geometric
ZIGP: zero-inflated generalized Poisson
MDD: Major Depressive Disorder
BIC: Bayesian Information Criteria
IRR: Incident Rate Ratio

## References

[1] World Health Organization. Depression 2018 [Available from: https://www.who.int/en/news-room/fact-sheets/detail/Depression. Accessed 28 Sept 2021.

[2] Curran C, Knapp M, McDaid D, Tómasson K, Group M (2007). Mental health and employment: an overview of patterns and policies across Western Europe. J Ment Health.

[3] de Macedo-Soares MB, Moreno RA, Rigonatti SP, Lafer B (2005). Efficacy of electroconvulsive therapy in treatment-resistant bipolar disorder: a case series. J ECT.

[4] Modabber-Nia M-J, Shodjai-Tehrani H, Moosavi S-R, Jahanbakhsh-Asli N, Fallahi M (2007). The prevalence of depression among high school and preuniversity adolescents: Rasht, northern Iran. Arch Iran Med.

[5] Depression W (2017). Other common mental disorders: global health estimates.

[6] Hasler G (2010). Pathophysiology of depression: do we have any solid evidence of interest to clinicians?. World Psychiatr.

[7] Kessler RC, Berglund P, Demler O, Jin R, Koretz D, Merikangas KR, Rush AJ, Walters EE, Wang PS (2003). The epidemiology of major depressive disorder: results from the National Comorbidity Survey Replication (NCS-R). Jama.

[8] McKnight PE, Kashdan TB (2009). The importance of functional impairment to mental health outcomes: a case for reassessing our goals in depression treatment research. Clin Psychol Rev.

[9] Katon W (2009). The impact of depression on workplace functioning and disability costs. Am J Manag Care.

[10] Kennedy SH, Eisfeld BS, Cooke RG (2001). Quality of life: an important dimension in assessing the treatment of depression?. J Psychiatr Neurosci JPN.

[11] Sadock B, Sadock V, Ruiz P (2015). Summary of psychiatry: behavioral sciences clinical psychoiatry.

[12] Mullen S (2018). Major depressive disorder in children and adolescents. Mental Health Clin.

[13] Kaplan BJ (2016). Kaplan and Sadock’s synopsis of psychiatry. Behavioral sciences/clinical psychiatry. Tijdschrift voor Psychiatrie.

[14] Frank E, Kupfer DJ, Perel JM, Cornes C, Jarrett DB, Mallinger AG, Thase ME, McEachran A, Grochocinski VJ (1990). Three-year outcomes for maintenance therapies in recurrent depression. Arch Gen Psychiatr.

[15] Gonzales LR, Lewinsohn PM, Clarke GN (1985). Longitudinal follow-up of unipolar depressives: an investigation of predictors of relapse. J Consult Clin Psychol.

[16] Hollon SD, DeRubeis RJ, Evans MD, Wiemer MJ, Garvey MJ, Grove WM, Tuason VB (1992). Cognitive therapy and pharmacotherapy for depression: singly and in combination. Arch Gen Psychiatr.

[17] Keller MB, Lavori PW, Lewis CE, Klerman GL (1983). Predictors of relapse in major depressive disorder. Jama.

[18] Maj M, Veltro F, Pirozzi R, Lobrace S, Magliano L. Pattern of recurrence of illness after recovery from an episode of major depression: a prospective study. Am J Psychiatr. 1992. 10.1176/ajp.149.6.795 1590496

[19] Roy-Byrne P, Post RM, Uhde TW, Porcu T, Davis D (1985). The longitudinal course of recurrent affective illness: life chart data from research patients at the NIMH. Acta Psychiatr Scand.

[20] Burcusa SL, Iacono WG (2007). Risk for recurrence in depression. Clin Psychol Rev.

[21] Judd LL (1997). The clinical course of unipolar major depressive disorders. Arch Gen Psychiatr.

[22] Balkrishnan R, Joish VN, Yang T, Jayawant SS, Mullins CD (2008). The economic burden associated with SSRI treatment failure in a managed care population. J Med Econ.

[23] Fostick L, Silberman A, Beckman M, Spivak B, Amital D (2010). The economic impact of depression: resistance or severity?. Eur Neuropsychopharmacol.

[24] Judd LL, Akiskal HS, Zeller PJ, Paulus M, Leon AC, Maser JD, Endicott J, Coryell W, Kunovac JL, Mueller TI, Rice JP, Keller MB (2000). Psychosocial disability during the long-term course of unipolar major depressive disorder. Arch Gen Psychiatr.

[25] Skodol AE, Grilo CM, Keyes KM, Geier T, Grant BF, Hasin DS (2011). Relationship of personality disorders to the course of major depressive disorder in a nationally representative sample. Am J Psychiatr.

[26] Kupfer DJ, Frank E, Wamhoff J. Mood disorders: Update on prevention of recurrence. In: Mundt C, Goldstein MJ, editors. Interpersonal factors in the origin and course of affective disorders. London, England: Gaskell/Royal College of Psychiatrists; 1996. pp. 289–302.

[27] Perris C. The distinction between unipolar and bipolar mood disorders: a 25-year perspective. L'Encéphale: Revue de psychiatrie clinique biologique et thérapeutique. 1992;18(1):9–13. 1600919

[28] Hasin DS, Sarvet AL, Meyers JL, Saha TD, Ruan WJ, Stohl M, Grant BF (2018). Epidemiology of adult DSM-5 major depressive disorder and its specifiers in the United States. JAMA Psychiatr.

[29] Trivedi MH, Rush AJ, Wisniewski SR, Nierenberg AA, Warden D, Ritz L, Norquist G, Howland RH, Lebowitz B, McGrath PJ, Shores-Wilson K, Biggs MM, Balasubramani GK, Fava M, STAR*D Study Team (2006). Evaluation of outcomes with citalopram for depression using measurement-based care in STAR* D: implications for clinical practice. Am J Psychiatr.

[30] Cheung A, Mayes T, Levitt A, Schaffer A, Michalak E, Kiss A, Emslie G (2010). Anxiety as a predictor of treatment outcome in children and adolescents with depression. J Child Adolesc Psychopharmacol.

[31] Barkow K, Maier W, Üstün TB, Gänsicke M, Wittchen H-U, Heun R (2003). Risk factors for depression at 12-month follow-up in adult primary health care patients with major depression: an international prospective study. J Affect Disord.

[32] Weinberger MI, Sirey JA, Bruce ML, Heo M, Papademetriou E, Meyers BS (2008). Predictors of major depression six months after admission for outpatient treatment. Psychiatr Serv.

[33] Alwan A, Saeed K (2015). A new agenda for mental health in the eastern Mediterranean region. EMHJ East Mediterr Health J.

[34] Haghgoo A, Zoladl M, Afroughi S, Rahimian H, Mirzaee MS. Assessment of the Burden on Family Caregivers of Patients with Mental Disorders Hospitalized in Shahid Rajai Hospital in Yasuj, 2016. Assessment. 2017;5(2):39–44.

[35] Halvorsen M, Wang CE, Eisemann M, Waterloo K (2010). Dysfunctional attitudes and early maladaptive schemas as predictors of depression: a 9-year follow-up study. Cogn Ther Res.

[36] Saragoussi D, Touya M, Haro JM, Jönsson B, Knapp M, Botrel B, Florea I, Loft H, Rive B (2017). Factors associated with failure to achieve remission and with relapse after remission in patients with major depressive disorder in the PERFORM study. Neuropsychiatr Dis Treat.

[37] Gilman SE, Kawachi I, Fitzmaurice GM, Buka SL (2003). Socio-economic status, family disruption and residential stability in childhood: relation to onset, recurrence and remission of major depression. Psychol Med.

[38] Vuong QH (1989). Likelihood ratio tests for model selection and non-nested hypotheses. Econometrica: J Econometric Soc.

[39] Sharareh P, Leili T, Abbas M, Jalal P, Ali G (2020). Determining correlates of the average number of cigarette smoking among college students using count regression models. Sci Rep.

[40] Wainwright N, Surtees PG (2002). Childhood adversity, gender and depression over the life-course. J Affect Disord.

[41] Amiri S (2021). Unemployment associated with major depression disorder and depressive symptoms: A systematic review and meta-analysis. International journal of occupational safety and ergonomics.

[42] McLeod JD, Kessler RC, Landis KR (1992). Speed of recovery from major depressive episodes in a community sample of married men and women. J Abnorm Psychol.

[43] Stegenga BT, Geerlings MI, Torres-González F, Xavier M, Švab I, Penninx BW, Nazareth I, King M (2013). Risk factors for onset of multiple or long major depressive episodes versus single and short episodes. Soc Psychiatry Psychiatr Epidemiol.

[44] Zamanian Z, Riaei S, Kaveh N, Khosravani A, Sayadi M, Moemenbellah-Fard MD (2016). The prevalence of Depression and its associated factors among students at Shiraz University of Medical Sciences in 2012. J Health Sci Surveill Syst.

[45] Chevalier A, Feinstein L (2005). The causal effect of education on depression, 2004.

[46] Teraishi T, Hori H, Sasayama D, Matsuo J, Ogawa S, Ishida I, Nagashima A, Kinoshita Y, Ota M, Hattori K, Higuchi T, Kunugi H (2015). Personality in remitted major depressive disorder with single and recurrent episodes assessed with the temperament and character inventory. Psychiatry Clin Neurosci.

[47] Pouretemad HR, Naghavi HR, Malekafzali H, Noorbala AA, Davidian H, Ghanizadeh A, et al. Prevalence of Mood Disorders in Iran. Iran J Psychiatry. 2006;1(2):59–64.

[48] Brown GW, Moran P (1994). Clinical and psychosocial origins of chronic depressive episodes. Br J Psychiatr.

[49] Brown GW, Moran P, Harris T, Hepworth C, Robinson R (1994). Clinical and psychosocial origins of chronic depressive episodes: II: a patient enquiry. Br J Psychiatr.

[50] Hynninen KMJ, Breitve MH, Wiborg AB, Pallesen S, Nordhus IH (2005). Psychological characteristics of patients with chronic obstructive pulmonary disease: a review. J Psychosom Res.

[51] Spijker J, de Graaf R, Bijl RV, Beekman AT, Ormel J, Nolen WA (2004). Determinants of persistence of major depressive episodes in the general population. Results from the Netherlands mental health survey and incidence study (NEMESIS). J Affect Disord.

[52] Geerlings SW, Beekman AT, Deeg DJ, Van Tilburg W (2000). Physical health and the onset and persistence of depression in older adults: an eight-wave prospective community-based study. Psychol Med.

[53] Perlis RH, Nierenberg AA, Alpert JE, Pava J, Matthews JD, Buchin J, Sickinger AH, Fava M (2002). Effects of adding cognitive therapy to fluoxetine dose increase on risk of relapse and residual depressive symptoms in continuation treatment of major depressive disorder. J Clin Psychopharmacol.

[54] Rucci P, Frank E, Calugi S, Miniati M, Benvenuti A, Wallace M, Fagiolini A, Maggi L, Kupfer DJ, Cassano GB (2011). Incidence and predictors of relapse during continuation treatment of major depression with SSRI, interpersonal psychotherapy, or their combination. Depression and Anxiety.

[55] Hardeveld F, Spijker J, De Graaf R, Hendriks SM, Licht CM, Nolen WA (2013). Recurrence of major depressive disorder across different treatment settings: results from the NESDA study. J Affect Disord.

[56] Onaemo VN, Fawehinmi TO, D’Arcy C. Alcohol use disorder and the persistence/recurrence of major Depression. Can J Psychiatr. 2020. 10.1177/0706743720923065.

[57] Kendler KS, Neale MC, MacLean CJ, Heath AC, Eaves LJ, Kessler RC (1993). Smoking and major depression: a causal analysis. Arch Gen Psychiatr.

