# Supplementary material for: Identifying factors associated with the hospital readmission rate among patients with major depressive disorder
Source: BMC Psychiatry. 2021 Nov 1;21:542. doi: 10.1186/s12888-021-03559-7 (PMC8561957; doi:10.1186/s12888-021-03559-7)
Supplement: Supplementary file 1 — Additional file 1. Count regression models. [file 12888_2021_3559_MOESM1_ESM.docx]

Supplementary file

Count regressions

There are several regression models for analyzing count data including Poisson and Negative binomial (NB), generalized Poisson (GP),and exponentiated exponential geometric (EEG) as well as their zero-inflated counterparts (ZIP, ZINB, ZIGP, ZIEEG). Because there is no model with the best fitting for all data, there is a need to assess different models for a data set. So, selecting a model with the best goodness-of-fit based on different criteria is of great importance

*Poisson regression:* The Poisson probability distribution is as follows:

$f\left(y; \lambda\right)=\frac{e^{-\lambda}\lambda^{y}}{y!} y=0,1,2,3,...$ (1)

with $E(Y)=Var(y)=\lambda$, where $\lambda$ stands for the mean and variance of the response variable. In order to investigate the effect of explanatory variables, the canonical link (here logarithm of $\lambda$) is related to covariates through $\log(\lambda)=x^{'}\beta$.

*Negative binomial regression:* The [probability mass function](https://en.wikipedia.org/wiki/Probability_mass_function) of the negative binomial distribution is as follows:

$f(y;\lambda,\alpha)=\frac{\Gamma(y+\frac{1}{\alpha})}{\Gamma(\frac{1}{\alpha})\Gamma(y+1)}\left(\frac{1}{\left(1+\alpha\lambda\right)}\right)^{\frac{1}{\alpha}}\left(1-\frac{1}{\left(1+\alpha\lambda\right)}\right)^{y} y=0,1,2,...$ (2)

with mean and variance of $E\left(y\right)=\lambda$ and $V\left(y\right)=\lambda+(\alpha\lambda^{2})$ respectively. The canonical link function of the NB is $\log(\lambda)=x^{'}\beta$. The parameter $\alpha$ is called dispersion (over-dispersion) parameter ^34^.

*The Generalized Poisson regression model:* A distribution most often used for count data that has over/under dispersion is the Generalized Poisson distribution. This distribution is a generalization of the Poisson distribution. The probability distribution is as follows:

$f\left(y_{i}; \mu_{i}, r\right)=\left(\frac{\mu_{i}}{1+r\mu_{i}}\right)^{y_{i}}\frac{\left(1+ry_{i}\right)^{y_{i}-1}}{y_{i}!}\exp\left[\frac{-\mu_{i}\left(1+ry_{i}\right)}{1+r\mu_{i}}\right] y_{i}=0, 1, 2,...$

This model has two parameters $\mu_{i}$and $r$. $r$ is the distribution parameter. When$r\to0$, this model becomes a Poisson model, if $r>0$, it shows over-dispersion and if $r<0$, it shows under-dispersion. The mean and variance of this distribution are as follows:

$$\begin{matrix} E\left(y_{i}\right)=\mu_{i} \\ Var\left(y_{i}\right)=\mu_{i}\left(1+r\mu_{i}\right)^{2} \end{matrix}$$

A log link function for linking $\mu_{i}$ to a linear predictor $X_{i}$is as follows:

$$\log(\mu_{i})=X_{i}^{T}\beta$$

where $X_{i}$the vector shows the explanatory variables for the individual $\mathbf{(1,X}_{i1}\mathbf{,X}_{i2}\mathbf{,...,X}_{\mathbf{ip}}\mathbf{)}^{\mathbf{T}}$ and $\mathbf{\beta=(\beta}_{\mathbf{0}}\mathbf{,\beta}_{1}\mathbf{,\beta}_{2}\mathbf{,...,\beta}_{\mathbf{p}}\mathbf{)}^{\mathbf{T}}$ the vector of regression coefficients, p represents the number of predictor variables (38).

*Exponentiated-exponential geometric regression*: The exponentiated-exponential distribution is a unimodal and right-skewed distribution. The probability function of Y_i_ with EEG distribution is given as follows:

$f\left(y; \theta, c\right)=\left(1-\theta^{y+1}\right)^{c}-\left(1-\theta^{y}\right)^{c} y=0, 1, 2, ...$ (4)

where c > 0 (c affects the shape of the distribution and over/under dispersion; so that the values smaller ≤ 2 is related to the over-dispersion, while the values greater than 2 are related to both over/under/equi-dispersed distributions) and$0<p^{\lambda}=\theta<1$. This distribution does not have a mean and variance in a closed-form. Therefore, Famoy et al suggested that the regression problem should be handled through $\theta\left(x_{i}\right)=\theta_{i}=f\left(x_{i}, \beta\right)=e^{x'_{i}\beta}/1+e^{x'_{i}\beta}$ function ^33^.

*Zero-Inflated models*: Sometimes, count data consist of many zeros that cannot be handled using the above distributions. All distributions of Poisson, NB, GP, and EEGR can be considered as mixture models called zero-inflated (ZI) models to account for the excess zero counts. The general form of ZI models which is as follows is based on logistic regression (typically with a logit link) to predict which class the zero belongs to:

(5)

where f(y) stands for the count distribution and the is the uncertainty parameter (mixing proportion).
